# Supplementary material for: Dichotomy between the humoral and cellular responses elicited by mRNA and adenoviral vector vaccines against SARS-CoV-2
Source: medRxiv. 2021 Sep 21:2021.09.17.21263528. Preprint. [Version 1] doi: 10.1101/2021.09.17.21263528 (PMC8475964; doi:10.1101/2021.09.17.21263528)

medRxiv preprint doi: https://doi.org/10.1101/2021.09.17.21263528; this version posted September 21, 2021. The copyright holder for this preprint (which was not certified by peer review) is the author/funder, who has granted medRxiv a license to display the preprint in perpetuity.

It is made available under a CC-BY 4.0 International license .

Supplemental Figure Legend

Fig. S1. Gating strategy for SARS-CoV-2 S1 RBD-specific memory B cells. (**A**) Physical parameters; (**B**) Exclusion of dead cells and non-B cells ($CD14^+$, $CD3^+$, $CD4^+$, $CD16^+$); (**C**) $CD19^+CD20^{Lo}$ B cells were further gated to distinguish (**D**) RBD-specific B cells based on dual labeling in the same staining tube with two fluorescent RBD tetramers separately conjugated with Alexa Fluor 647 and BV421.

Figure S1

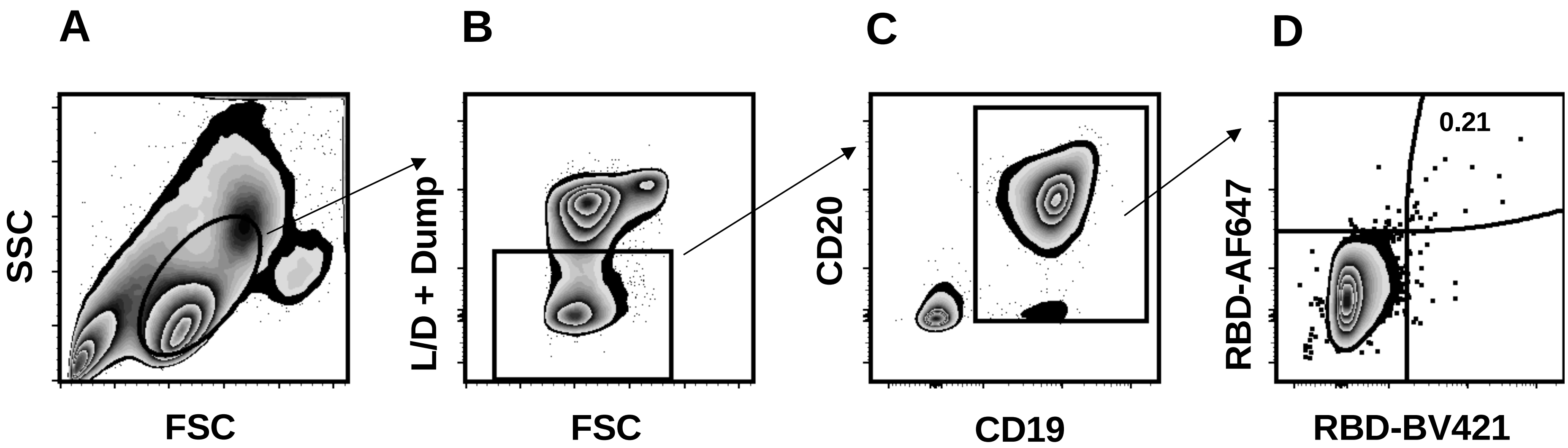

Supplement: 1 [file NIHPP2021.09.17.21263528V1-supplement-1.pdf]
